# Extraction of Inter-Aural Time Differences Using a Spiking Neuron Network Model of the Medial Superior Olive

**DOI:** 10.3389/fnins.2018.00140

**Published:** 2018-03-06

**Authors:** Jörg Encke, Werner Hemmert

**Affiliations:** Bioanaloge-Informationsverarbeitung, Department of Electrical and Computer Engineering, Technical University Munich, Munich, Germany

**Keywords:** spatial hearing, medial superior olive, computational model, artificial neural network, binaural model

## Abstract

The mammalian auditory system is able to extract temporal and spectral features from sound signals at the two ears. One important cue for localization of low-frequency sound sources in the horizontal plane are inter-aural time differences (ITDs) which are first analyzed in the medial superior olive (MSO) in the brainstem. Neural recordings of ITD tuning curves at various stages along the auditory pathway suggest that ITDs in the mammalian brainstem are not represented in form of a Jeffress-type place code. An alternative is the hemispheric opponent-channel code, according to which ITDs are encoded as the difference in the responses of the MSO nuclei in the two hemispheres. In this study, we present a physiologically-plausible, spiking neuron network model of the mammalian MSO circuit and apply two different methods of extracting ITDs from arbitrary sound signals. The network model is driven by a functional model of the auditory periphery and physiological models of the cochlear nucleus and the MSO. Using a linear opponent-channel decoder, we show that the network is able to detect changes in ITD with a precision down to 10 μs and that the sensitivity of the decoder depends on the slope of the ITD-rate functions. A second approach uses an artificial neuronal network to predict ITDs directly from the spiking output of the MSO and ANF model. Using this predictor, we show that the MSO-network is able to reliably encode static and time-dependent ITDs over a large frequency range, also for complex signals like speech.

## 1. Introduction

Our remarkable sound localization acuity relies on the ability of the auditory system to decode the arrival time and intensity difference between the ear canal signals into information about the direction of sound sources. In mammals, the primary nucleus to extract fine structure interaural time differences (ITDs) is the medial superior olive (MSO), while the interaural level differences (ILDs) are extracted primarily at the lateral superior olive (LSO) (Grothe et al., 2010). The MSO neurons detect fine-structure ITDs by acting as coincidence detectors receiving excitatory inputs from both hemispheres. The existence of such neurons was already hypothesized by Jeffress (1948), who proposed an array of coincident detectors to be arranged along a neural delay line. In this hypothesis, each neuron would respond maximally to a specific ITD (best-ITD)—generating a topographical mapping of time differences within the nucleus. Later, such a circuit was found in the nucleus laminaris of birds like the barn owl (Carr and Konishi, 1988). However, more recent measurements of mammalian inferior colliculus (IC) and MSO neurons in gerbils (Brand et al., 2002) or guinea pigs (McAlpine et al., 2001) revealed broadly-tuned neurons, of which the majority had their best-ITDs at the border or even outside of the animals physiological range. This observation is inconsistent with place-code theory, which would require a vast amount of narrowly-tuned neurons with their best-ITDs distributed within the physiological range. One alternative ITD-coding mechanism is based on the comparison of firing rates between the nuclei in the two hemispheres. This mechanism has consequently been called the opponent-channel (Magezi and Krumbholz, 2010), count-comparison (Colburn and Durlach, 1978), or hemifield (Stecker et al., 2005) model. The opponent-coding model is in agreement with both observations, the wide tuning curves and the large best-ITDs (McAlpine and Grothe, 2003). There is also evidence that overall sound localization (Stecker et al., 2005; Briley et al., 2012) as well as specifically ITD-coding in the human auditory cortex is based on an opponent coding mechanism (Salminen et al., 2010). Lesion studies in cats showed that unilateral lesions at the level of the central auditory system (Jenkins and Masterton, 1982) as well as in cortical regions (Malhotra et al., 2004) mainly resulted in deficits localizing sounds from locations contralateral of the lesion. These results lead Jenkins and Masterton (1982) to conclude that each auditory-hemifield is represented solely in the respective contralateral hemisphere, which would contradict the opponent coding mechanism. One problem with applying this interpretation to ITD processing is that both studies used broad-band stimuli so that ITDs and ILDs, as well as spectral and monaural cues were available to localize the sound source this makes it difficult to draw conclusions about the representation of the individual cue. An alternative to the opponent-channel code, which uses the summed response of the neurons within each of the two hemispheres, is the population decoder that instead uses the individual response of each neuron for decoding. Based neuronal recordings of neurons in the IC, Goodman et al. (2013) and Day and Delgutte (2013) both proposed population decoders and showed that these decoders could outperform a two-channel decoder. On the other hand, Harper et al. (2014) used an optimal coding approach to show that ITDs in low-frequency signals would be best represented by a two-channel code. Additionally, results from psychoacoustic lateralization experiments using pure-tone adapter stimuli with fixed ITDs showed, that adaptation influences lateralization at ITDs not only close to that of the adapter but within the whole hemisphere (Phillips et al., 2006), which is more in line with an opponent-channel code.

The aforementioned remarkable sound localization ability has inspired numerous researchers to create computational binaural models. Most of the existing binaural models are phenomenological implementations of the delay-line principle proposed by Jeffress (1948), which have been tuned to successfully predict data from human psychoacoustics (Lindemann, 1986). Some more recent models were implemented following the opponent-coding mechanism (Pulkki and Hirvonen, 2009; Dietz et al., 2011; Takanen et al., 2014). Even though these models closely follow the functionality of the neuronal sound localization pathway, they provide only a phenomenological description of the processing stages. On the other hand, several biophysical models of MSO neurons have been published as well (Brughera et al., 1996, 2013; Zhou et al., 2005; Lehnert et al., 2014), but there are only a few biophysical models covering the complete neuronal circuit. Wang et al. (2013) used a circuit containing a model of the auditory periphery as well as spiking models of the MSO and LSO and a simplified IC model to investigate the sensitivity of IC neurons to envelope ITDs in high-frequency sounds. Due to the focus on high-frequency sounds where ITDs are extracted from the envelope of the sound signal instead of its fine structure (Nuetzel and Hafter, 1976), Wang et al. (2013) did not include any source for a shift in best-ITD and also neglected inhibitory inputs to the MSO. Glackin et al. (2010) presented a spiking neural network (SNN) constructed from leaky integrate-and-fire models of the CN and MSO nuclei. In disagreement with newer physiological studies, the SNN was constructed as a Jeffress-type delay-line decoder. Glackin et al. (2010) trained the network to localize the sounds using spike-timing-dependent plasticity learning rules.

To our knowledge, none of the previous models combined an SNN approach with the concept of opponent-coding to investigate ITD sensitivity. Brughera et al. (2013) presented a single spiking neuron model of the MSO to investigated ITD sensitivity, but used a periodic Poisson-like process as an input to the MSO. This limits the model to simple pure-tone-like scenarios while also neglecting any non-linear processing of the auditory periphery. To that end, we present here a new binaural model based on biophysical spiking neuron models of the mammalian MSO circuit. We show that a simple linear hemisphere decoder applied to the output of the model is sufficient to encode ITDs in tones with a precision that matches human performance. Furthermore, we show how the model in conjunction with a simple artificial neural network can decode ITDs from broadband signals, including complex signals like speech.

## 2. Results

### 2.1. Model structure

**Figure 1:**
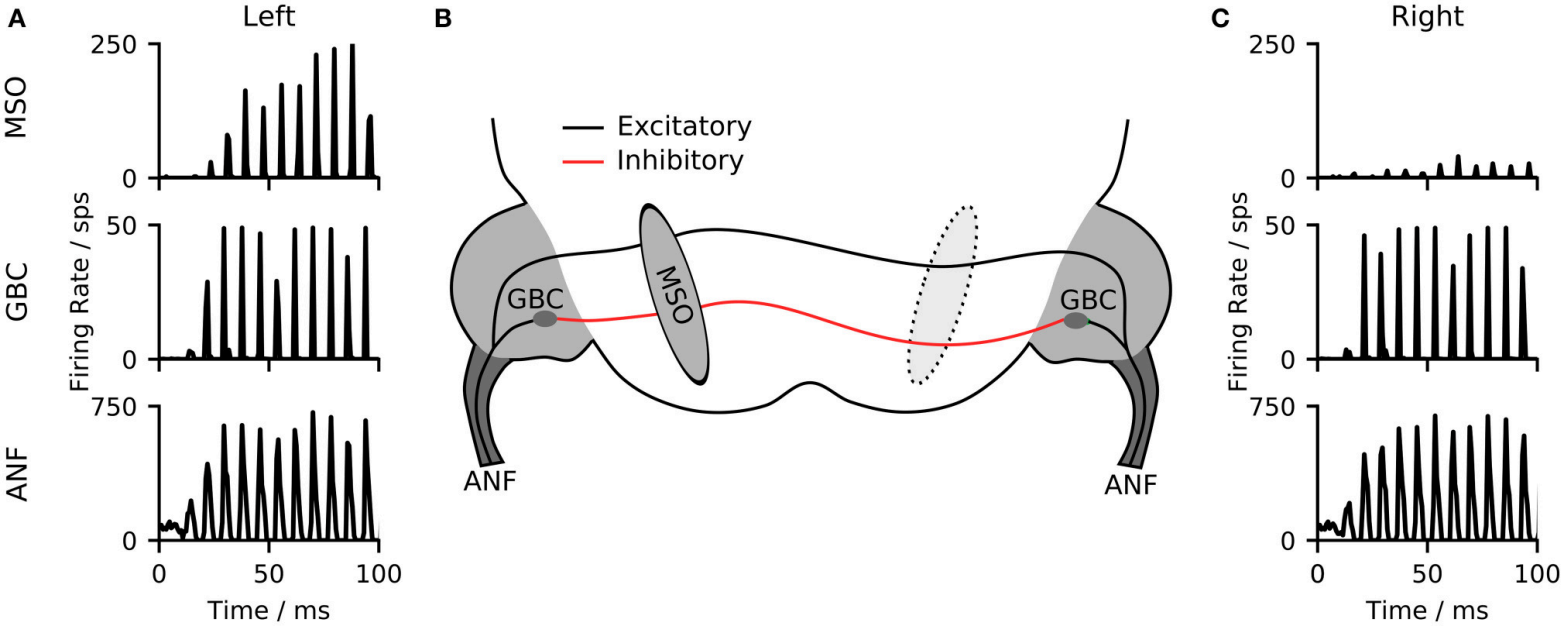
**(A,C)** Poststimulus time histograms (750 μs bin size) of the responses of the three model stages to a 100 ms long pure tone. **(B)** The model network contains three stages. A model of the auditory periphery (ANF), A model of the globular bushy cells in the cochlear nucleus (GBC), and the model of the medial superior olive (MSO).

The primary mammalian MSO neurons receive excitatory inputs from spherical bushy cells (SBCs) as well as inhibitory inputs from the globular bushy cells (GBCs) of the cochlear nuclei in both hemispheres. Inhibitory inputs are being relayed via the trapezoid body (TB) (see Grothe et al., 2010 for an overview). Both SBCs and GBCs are directly excited by auditory nerve fibers (ANFs). GBCs in particular, but also SBCs have been found to enhance phase locking of the neuronal inputs (Joris et al., 1994; Dehmel et al., 2010). Our model consists of three stages, a model of the auditory periphery, a population of globular bushy cells and a population of MSO neurons (see Figure 1). For simplicity, SBC as well as the TB nuclei, were reflected as direct relays of the ANF signals so that our MSO model receives direct excitatory input from the ANF and inhibitory inputs from GBCs of both hemispheres (see section 4 for details on the implementation). In practice, our model takes digitized binaural signals as input and processes them first through the peripheral hearing models of the left and right ears. The peripheral model consists of a middle-ear compensation filter, a non-linear model of the basilar membrane and a functional model of the neural transduction of the inner hair cell and auditory nerve fibers (Zilany et al., 2014). All ANFs were modeled as high spontaneous rate units. The spike timings of the peripheral hearing models were then used as input to the biophysical neuron models. As a consequence of the direct excitation by ANF fibers, the frequency responses of both MSO and GBCs resemble that of the ANFs from the peripheral hearing model (see Figure S1).

As an example of the output from the different model stages, Figures 1A,C illustrate the outputs of ANFs, GBCs, and the MSO of the two hemispheres for a left-leading (150 μs ITD) 125 Hz pure-tone input. The ANFs of both hemispheres show a phase-locked response to the input stimulus. This phase-locked response is sharpened by the population of GBC neurons. The MSO neurons of the two hemispheres respond with different firing rates depending on the delay between the signals delivered to the left and right ear.

**Figure 2:**
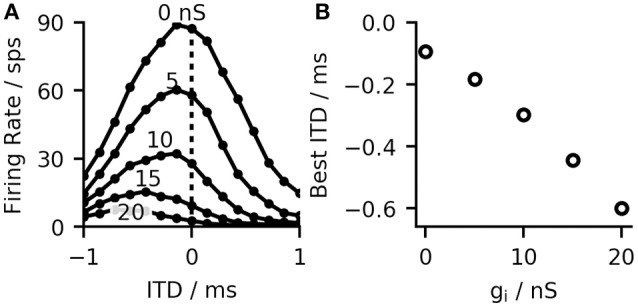
**(A)** MSO ITD-rate functions (calculated for 15 ITDs in the range ±1 ms) for the right hemisphere of the model at different inhibitory conductivities ĝ_*syn, i*_ **(B)** Increased inhibition, reduces the overall firing rate and shifts the best-ITD toward more contra-lateral leading ITDs. Without inhibition the best-ITD equals the predefined shift of 100 μs.

Most MSO neurons of gerbils show bell-shaped ITD-rate functions with their maximum (best-ITD) located outside of the animals physiological range (Brand et al., 2002). There has been much debate about the origin of this shift ranging from intra-cochlear delays (Joris et al., 2006) over asymmetric synaptic currents (Jercog et al., 2010) to effects of the recent stimulus history (Franken et al., 2015). Our model is based on the effect described by Brand et al. (2002) and Pecka et al. (2008), who showed that blocking of the inhibitory inputs results in a shift of the best-ITD toward zero. Measurements in gerbil brain slices have also shown that inhibitory inputs to the MSO precede the excitatory inputs in time (Roberts et al., 2013). Using conduction clamp measurements, Myoga et al. (2014) showed that the relative timing of inhibitory to excitatory inputs to the MSO can delay or advance the peak of the excitatory post-synaptic potential (EPSP) and consequently, affect the best ITD of the neurons. Our model is consistent with these findings. In agreement with Brand et al. (2002) and Pecka et al. (2008), the best-ITD shifted toward zero when simulating the effect of blocked inhibition by reducing the inhibitory synaptic strength (see Figures 2A,B). Similarly, and in accordance with Myoga et al. (2014), we could shift the best-ITD of the MSO model by adjusting the delay of contra- and ipsilateral inhibitory inputs. For the model used in later evaluations, we optimized both arrival times to obtain a maximal shift of the best-ITD toward contra-leading ITDs. This optimization resulted in a delay of 0.6 ms for the contralateral inhibitory input and 0 ms for the ipsilateral input (both values relative to the timing of the excitatory input from the corresponding side). These values are in agreement with the timescales observed by both Myoga et al. (2014) and Roberts et al. (2013). The study by Pecka et al. (2008) showed a residual shift of the best-ITD even when the inhibitory inputs were blocked. This could be explained by fundamental physics as the axons connecting inputs from the contralateral hemisphere to the MSO have to span over a larger distance than the ones for ipsilateral inputs. We considered this observation by adding a constant delay of 100 μs to the contralateral excitatory and inhibitory inputs, which resulted in an additional shift of the best-ITD toward negative values (see Figure 2B).

### 2.2. Decoding ITD information from the neuronal responses

**Figure 3:**
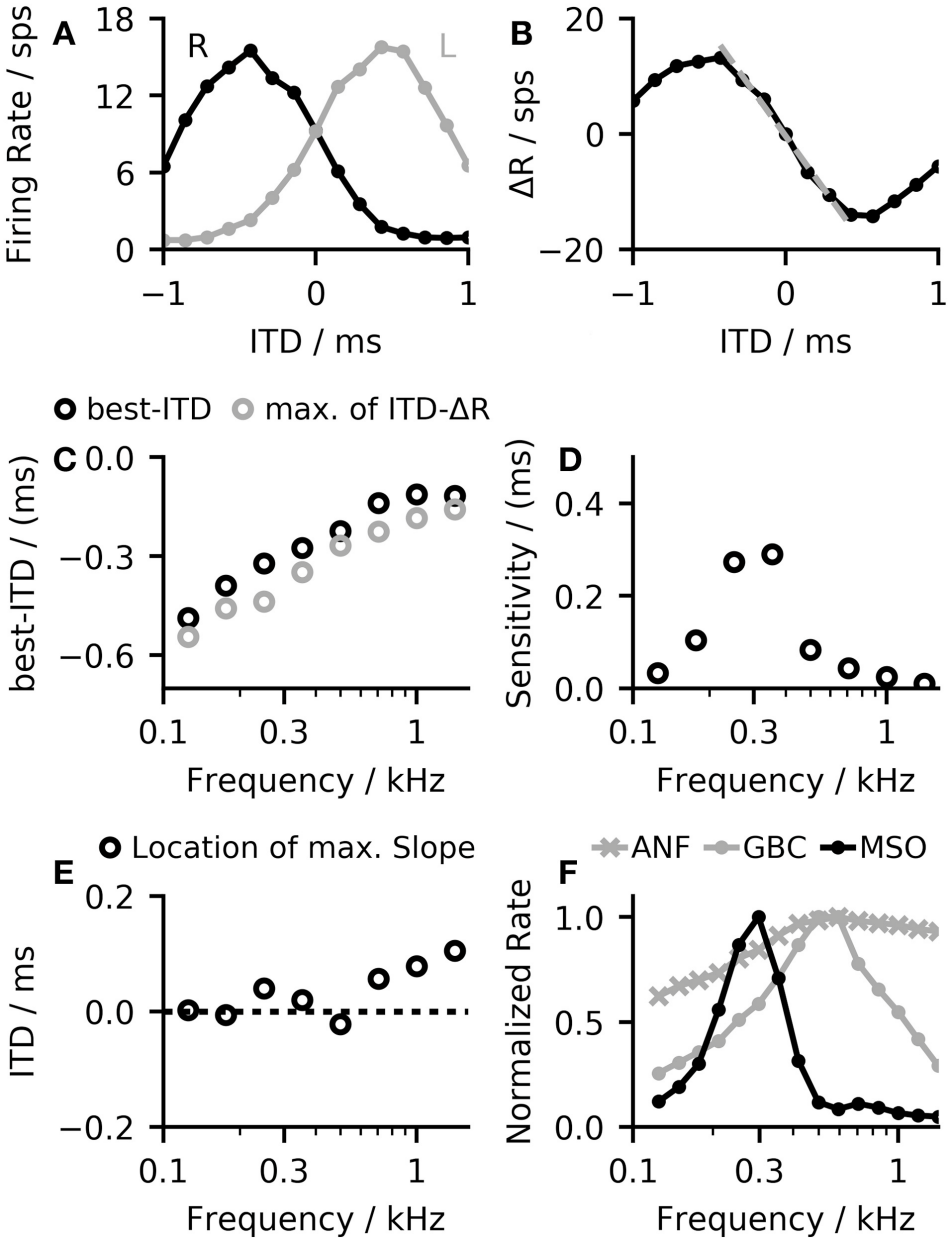
**(A)** The model demonstrates ITD-rate functions for the left and right MSO that are effectively mirrored around zero so that a shift in ITD from the center line leads to an inverse response of the two channels forming the basis for the opponent-channel code (Stecker et al., 2005). **(B)** A simple difference computation between the ITD-rate functions of the two hemispheres resulted in a nearly linear relationship around zero ITD (dashed line). **(C)** The shift in the best-ITD decreases with increasing best-frequency of the MSO neuron, reaching the predefined shift of 100 μs at about 1.4 kHz. **(D)** The slope of the rate difference curve around midline can be seen as a sensitivity to changes in ITD. This value changes with the best frequency. The sensitivity peaks at 300 Hz from where it decreases again toward higher frequencies. **(E)** The single hemisphere responses showed the largest change at or close to the midline and therefore maximizes the sensitivity of the linear-decoder. **(F)** Frequency-dependent normalized firing rates of the three neuron populations in the model in response to a 100 ms pure tone at 50 dB.

The opponent-coding theory is based on two populations of neurons, both firing maximally when the sound source is on the opposite side of the midline (Stecker et al., 2005). Figure 3A shows firing rates of the MSO model in both hemispheres to a stimulation with varying ITDs. The left MSO responds strongest when the stimulus was right-leading (positive ITD), while the right hemisphere responds strongest to a left-leading ITD (negative value). Consequently, a change in ITD from zero results in an increased firing of one MSO and a reduced firing of the other. A very basic decoder for the opponent-channel code can be constructed by subtracting the firing rates of the left MSO (*R*_*L*_) from the right MSO (*R*_*R*_). Around zero ITD, the calculated firing rate difference Δ*R* = *R*_*R*_−*R*_*L*_ shows an almost linear response to ITD changes (see Figure 3B). Due to the subtraction, this approach increases the slope around zero by a factor of two and consequently maximizes the sensitivity in this region. However, this approach is applicable only for ITDs for which the linear approximation is valid. The linear ITD region depends primarily on the location of the best-ITD (see Figure 3C) in the two hemispheres. When calculating ITD-rate functions for neurons with different best-frequency, the best ITD decreases with increasing sound frequency (see Figure 3C). The best-ITD is maximally 470 μs at 125 Hz and decreases to 110 μs at 1.4 kHz. The same trend of decreasing best-ITDs with increasing frequency has been found in *in-vivo* recordings of MSO neurons (Brand et al., 2002; Pecka et al., 2008) as well as in the IC (McAlpine et al., 2001). As aforementioned, this model mainly uses phase-locked inhibition to shift the best-ITD. This method relies on the slopes of the inhibitory post-synaptic potentials (IPSPs) of each phase (Myoga et al., 2014). At higher frequencies, the summation of individual IPSPs reduces the effectiveness in shifting the best-ITD (Roberts et al., 2013; Myoga et al., 2014), which is also seen in the model results. Experimental studies have shown that MSO and IC neurons exhibit a variety of different best-ITDs (McAlpine et al., 2001; Bremen and Joris, 2013), while in this model, all neurons with the same best frequency also show the same best-ITD. As this study does not use a population decoder but relies on the mean activity within each hemisphere, the single ITD-rate function can also be interpreted as the mean ITD-rate function of a single hemisphere.

The sensitivity of the linear-decoder to ITD changes is proportional to the slope of the ΔR function around zero ITD—a steeper slope results in larger changes. As the slope of the ΔR function around zero is twice the slope of a single hemisphere response, maximizing the slope of the single hemisphere will also result in a maximal slope of ΔR. Pecka et al. (2008) and McAlpine et al. (2001) both reported the maximal slope of single neuron responses to be located at or close to mid-line. In this model, responses to frequencies up to 700 Hz followed these findings (see Figure 3E). At higher frequencies, the location of the largest slope started to shifted away from midline as the best-ITD decreased faster than the width of the ITD-tuning function, which shifted the location of the largest slope toward positive ITDs. A second influencing factor on the sensitivity is the maximum firing rate of the MSO response—a higher rate of the single hemisphere responses will also result in a larger slope at midline. Figure 3F shows the frequency dependent normalized firing rates of all three neuron populations in the model. The firing rate of the MSO model is of course strongly influenced by the balance between the excitatory inputs from the ANFs and the inhibitory inputs from the GBCs but it is additionally modulated by changes of the spiking thresholds. MSO neurons have been found to exhibit subthreshold resonance (Remme et al., 2014; Mikiel-Hunter et al., 2016) which introduces frequency dependent thresholds. The MSO model used in this study exhibited a resonance frequency at about 260 Hz (see Figure S2) which is in agreement with the resonance frequencies found in electrophysiological studies (Remme et al., 2014; Mikiel-Hunter et al., 2016). The reduced spiking threshold around 260 Hz in combination with the dynamics of the synaptic inputs results in a peak in MSO response seen in Figure 3F, which also corresponds to the peak in sensitivity shown in Figure 3D.

**Figure 4:**
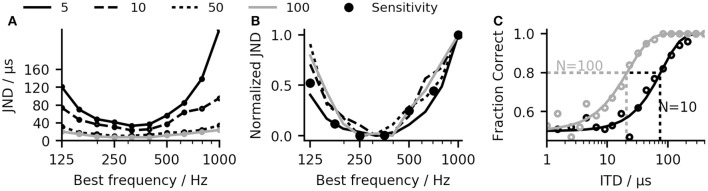
**(A)** Frequency-dependent just-noticeable differences (JNDs) calculated for subsets of 5, 10, 50, and 100 randomly chosen neurons among a population of 500 neurons. **(B)** When normalized to a region between 0 and 1, all JND curves overlap. **(C)** The JND values were calculated by fitting weibull-functions to the fraction correct values (see section 4).

While applying the linear-decoder does not directly result in an ITD estimate, it can be used to predict ITDs. The link between ITD and ΔR also allows for a direct comparison of the laterality of two signals with different ITDs without the necessity to map the MSO model response to the absolute ITD estimates. This highlights the difference between an absolute localization task, which requires the mapping of the auditory perception to a spatial measure and a relative comparison task where the relative location of one perception in comparison to a second perception is reported. In psychoacoustical experiments, the sensitivity to ITDs is often assessed by determining the just noticeable differences (JNDs) which describe the smallest change in ITD a subject can use to detect a change in lateralization between the two otherwise identical stimuli (Klumpp, 1956). Using the same method, we calculated JNDs for our network model using the linear-decoder (see section 4). In our model, the performance depends critically on the number of neurons composing the population, as the intrinsic stochasticity of the neuronal system loses its impact on the average firing rate when the population increases. To determine the influence of the population size on the performance of our model, JNDs were calculated separately for subsets of 5, 10, 50, and 100 randomly chosen neurons among a population of 500 neurons. Figure 4C shows exemplary psychometric curves derived for a population of 10 and 100 neurons. Figure 4A shows the result of the JND experiment for different pure tone stimuli. As expected, the predicted JND decreases when increasing the size of the population. The decrease in JND can be described by a $1/\sqrt{N}$ dependency, where *N* is the population size. The dependence is in line with the reduced effect of noise due to a larger population of neurons. If the JND thresholds are determined mainly by the noise of the system, they should also be reflected in the sensitivity described by the slope of the Δ*R* function. Figure 4B shows the JND curve as well as the inverse of the slope of Δ*R* with all values normalized to lie between 0 and 1. As expected, there is a good agreement between the normalized JND curves and the inverse of the slope, which confirms the aforementioned assumption that the detection threshold of the linear-decoder depends mainly on the slope of the rate-difference function around zero ITD.

**Figure 5:**
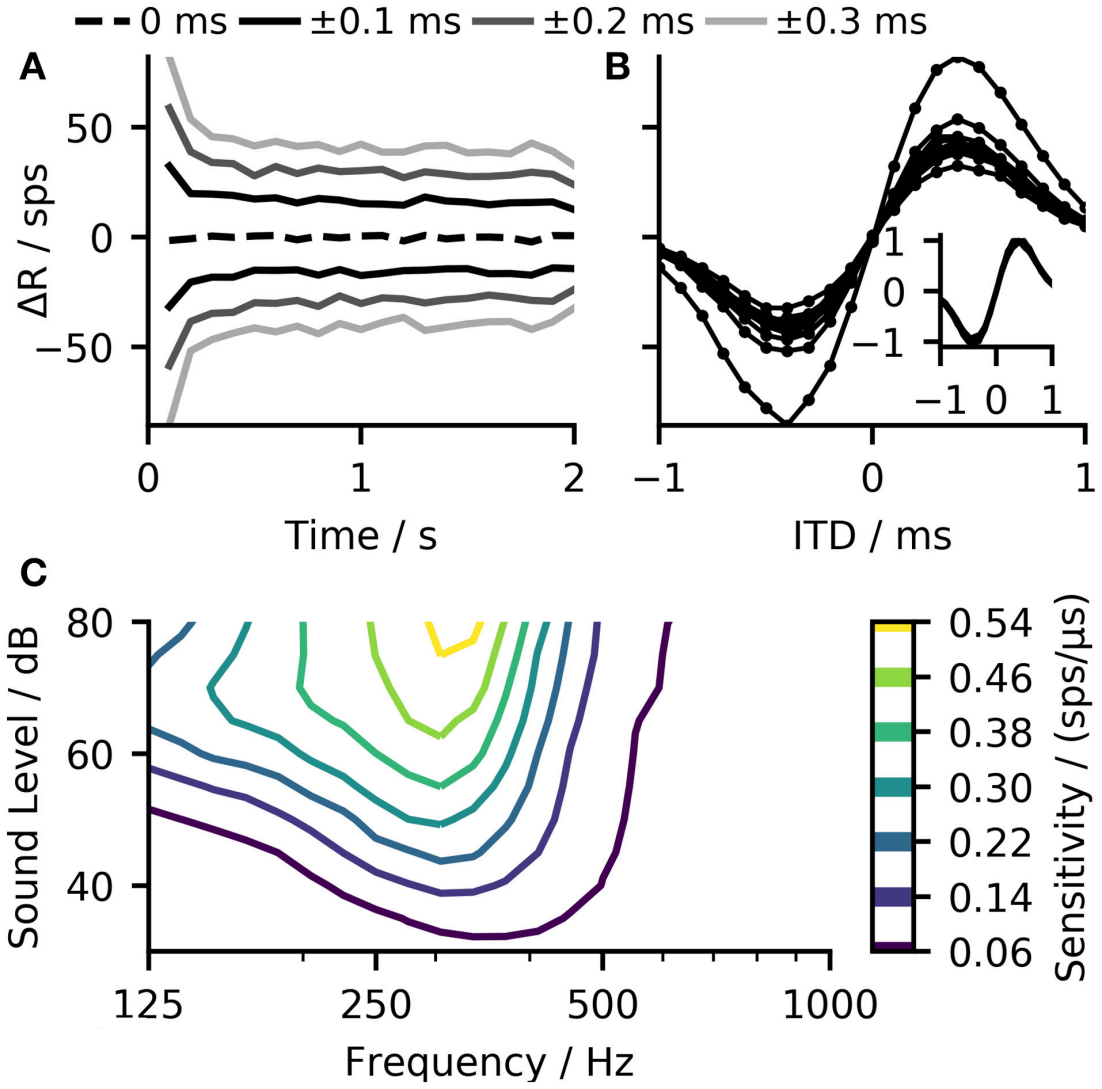
**(A)** The ΔR function (calculated for 100 ms bins) of 500 MSO neurons in response to a two second long 250 Hz pure tone with ITDs ranging from 0 to ±0.3 ms show a strong influence of the peripheral hearing model's adaptation on the MSO model output. **(B)** The same data as in **(A)** but shown in form of ITD-ΔR functions. Every function corresponds to one point in time evaluated for 20 ITDs in the range from −1 to 1 ms. The difference in the slope of these functions illustrates that adaptation influences the sensitivity of the linear-decoder. Normalization with respect to the maximal rate of each function could compensate for this influence (overlay) **(C)** The model displays strong variations in sensitivity with sound frequency and sound level.

One problem of such a linear-decoder is that the firing rates of the two MSO models depends not solely on the ITD, but also on other characteristics of the inputs to the MSO model. As the firing rate of the peripheral hearing model depend strongly on the sound pressure level, so will the output of the MSO model. To demonstrate such dependency, Figure 5C shows how the predicted sensitivity of our model varies with both frequency and level of the pure tone input. The ANFs also exhibit strong spike-rate adaptation (Smith, 1977) which, consequently affects the MSO response (Figure 5A). These variations could be compensated by normalizing the ITD-ΔR functions (overlay in Figure 5B) but this is not possible in practice as it would require *a priori* knowledge about the maximum firing rate of the ITD-ΔR function at each point in time. A much more practical approach is to compensate such non-linear dependencies using the information that is already encoded in the ANF firing rates.

The MSO exhibits a distinct tonotopic organization along its dorsoventral axis. As the neuronal populations along the axis differ in their characteristic frequency (Guinan et al., 1972), consequently, a given ITD decoder can specialize on decoding of ITDs within a specific frequency range. In addition, the non-linear and time-dependent output of the peripheral hearing process can be compensated by using direct knowledge about the firing rates of the ANF. However, implementing such corrections would require designing a complex multi-dimensional correction function. Artificial neuronal networks (ANN) have been proven to be quite successful in learning the behavior of highly nonlinear systems (Almeida, 2002), hence, they provide an appealing alternative to tedious manual construction of a correction function.

### 2.3. Artificial neuronal network predictor

We used a small multi-layer perceptron (MLP) to predict ITD values from the output of the SNN model by means of non-linear regression analysis. The regression is based on the average firing rates across the neuronal populations and predictions are calculated separately for each frequency band and time frame. The MLP was implemented using seven input nodes, one hidden layer with twenty nodes and two output nodes (for details see section 4). One of the MLP output nodes was used for the prediction task, while the second output was used to classify the reliability of the prediction based on the firing rates. This was deemed necessary to omit predictions for parts of the input signal, which did not contain enough energy in the given frequency band to enable robust predictions based on sufficient spiking activity.

**Figure 6:**
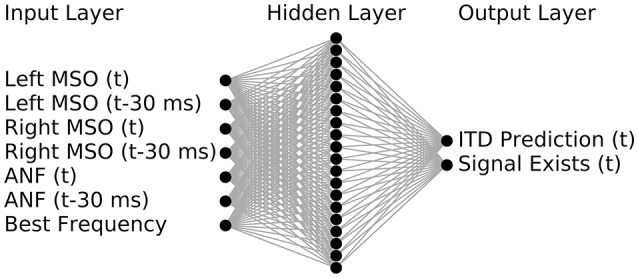
The ANN predictor was implemented using seven input nodes, one hidden layer with twenty nodes and two output nodes. Inputs denoted with (t) are firing rates within a given time period (typically 30 ms) for which the ITD should be predicted, while the ones denoted with (t-30 ms) are firing rates of the preceding time frame.

The inputs to the MLP were designed to consist of the firing rates from the MSO of the left and right hemisphere and the characteristic frequency of the neuron population (see Figure 6 for a schematic of the networks in- and outputs). As one of the main tasks of the predictor was to compensate for the influence of variations in the peripheral hearing model output, the MLP was also provided with a monolateral input of the ANF firing rate. All firing rates were provided as an average value computed over a predefined time period of 30 ms. This duration was chosen as it offered reasonably high temporal resolution and ensured that several periods of the phase locked input were included. In addition to the rates within the given time frame, we also provided firing rates of the previous time frame which reduced the noise in the predictions by effectively doubling of the time span that the network can employ in its predictions. The MLP was trained on 300 ms long pure tones (see section 4) covering the frequency range from 125 to 1,000 Hz so that predictions can be obtained for any stimuli within that range. For the following experiments we calculated predictions for 13 logarithmically spaced frequencies between 125 and 1,000 Hz.

**Figure 7:**
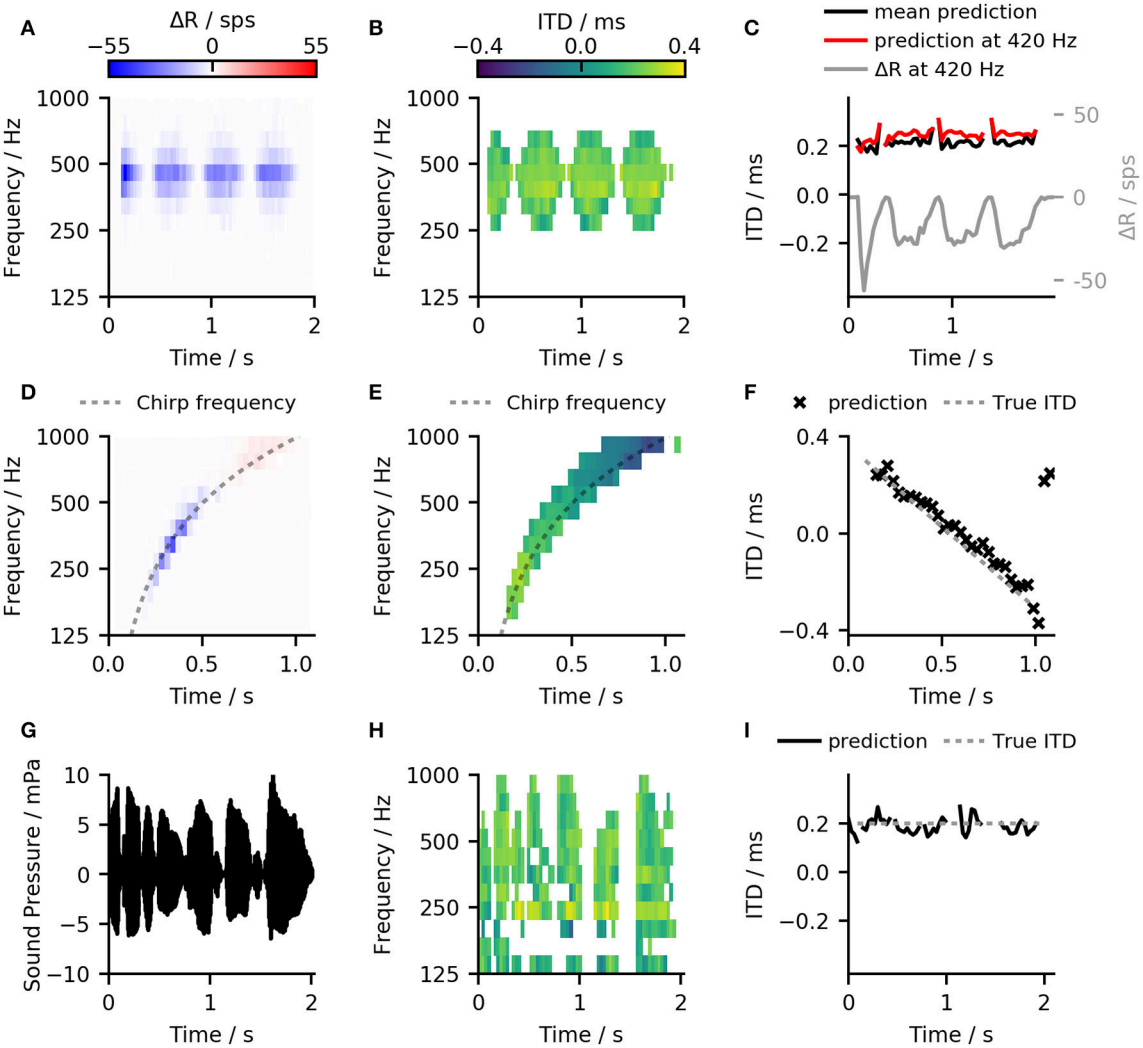
**(A)** Results of the linear-decoder for an amplitude-modulated tone with 400 Hz carrier frequency and a modulation rate of 2 Hz presented with an ITD of 200 ms. ΔR showed strong modulation with the modulation frequency of the sound as well as an influence of ANF adaptation **(B)** Results of the ANN-predictor predictor for the same signal as in **(A)**. The ANN was able to correct for the variations conveyed by the ANF inputs and to provide a stable prediction within the frequency bands from 250 Hz to 595 Hz. **(C)** The output of both, the ANN-predictor and the linear decoder for the amplitude modulated signal over time. Red: ANN predictions for the 420 Hz channel which was the closest to the stimulation frequency. Black: Mean over all predictions that were classified to contain a useful signal. Gray: Result of the linear-decoder in the 420 Hz frequency band. **(D,E)** Same plots as in **(A,B)** but for a linear, one-second long chirp ranging from 125 to 1 kHz where the ITD changed from −0.4 to 0.4 ms. **(F)** The ANN-predictor was able to follow the change in frequency as well as in ITD, deviating from the true value only at the end of the signal. **(G–I)** The ANN-predictor applied to a speech signal (German sentence “Britta gewann drei schwere Steine”) taken from the OLSA sentence test (Wagener et al., 1999).

Figures 7A–C compares the results of the ANN-predictor with those of the linear-decoder for an amplitude-modulated tone with 400 Hz carrier frequency and a modulation rate of 2 Hz. Since amplitude modulation is encoded in the firing rate of the ANF, it is also exhibited in the output of the linear-decoder (Figures 7A,C). On the other hand, the predictions from the ANN (Figure 7B) showed only minor deviations at the on- and offsets of each modulation cycle while largely compensating the strong onset response introduced by ANF's adaptation. Figure 7B shows only such predictions that the ANN classified to be reliable. In case of the amplitude-modulated signal, the frequency bands for which the ANN could predict ITDs are dependent on the phase of the modulation.

Omitting unreliable predictions enables the calculation of a general prediction across frequency bands. In case of the linear-decoder, zero output can correspond to two conditions—zero ITD and no signal. The employed method of omitting unreliable estimates is especially important for applying the ANN predictor to more complex signals that have several frequency components because the omission enables the ANN to predict ITDs without prior knowledge about the signal's frequency content. Figures 7D–F show examples of the ANN-predictor applied to a linear chirp. To demonstrate the ability of the predictor to follow changes both in frequency as well as in ITD, an additional phase shift was applied to the left ear signal. This phase shift was chosen to be proportional to an ITD-value that varied linearly from +300 to −300 μs. By calculating a cross-frequency prediction for every time frame, the ANN-predictor was able to follow the change in frequency as well in ITD (Figure 7C) deviating from the true value only in the last two time frames. As a final example, we show the ANN-predictor applied to a speech signal with a static ITD of 200 μs (Figures 7G–I). Again, the across-frequency estimation, combined with the omission of unreliable predictions allows the ANN-predictor to offer an accurate estimate of the ITD for the whole signal.

## 3. Discussion and conclusion

In this study, we presented a novel binaural model and used it to detect ITDs in arbitrary sound signals. In contrast to previous binaural models that used a phenomenological modeling approach (Pulkki and Hirvonen, 2009; Dietz et al., 2011), this study used biophysical neuron models based on the current knowledge about the function of the mammalian MSO. Some previous studies implemented similar SNN but either used a simplified auditory periphery and thus limited the application of the model to pure tones (Brughera et al., 2013), based their model on topologies that disagree with newer physiological studies (Glackin et al., 2010) or focused on ITDs in the stimulus envelope (Wang et al., 2013). Using two different extraction methods, we found that applying the opponent-coding mechanism to the output of the model enabled a robust extraction of ITDs even in complex signals.

### 3.1. Sensitivity of the linear-decoder

We have shown that a simple linear-decoder can detect ITDs from the outputs of the left and right MSO models with a sensitivity that reflects human performance in a discrimination task and depended on sound frequency. The sensitivity was mainly determined by the maximum firing rate of the MSO at a given frequency. Our MSO neurons showed a peak at approximately 300 Hz. To our knowledge, no such systematic variation of firing rate with frequency has been described, but Yin and Chan (1990) noted a similar characteristic in the response of high-frequency MSO neurons. They recorded the response of neurons that phase-locked to the envelopes of amplitude-modulated tones and also showed a peak in the response at a modulation rate of 300 Hz. In the model, the responses were influenced by the subthreshold resonance of MSO neurons, which is due to the dynamics of the low threshold potassium current (Mikiel-Hunter et al., 2016). This resonance would also explain the results by Yin and Chan (1990). An explanation why no similar result in the response of low-frequency MSO neurons has been described is that these measurements are limited to responses derived at the neurons' best-frequency so that any systematic variation between neurons with different best-frequencies could be masked by variations in the overall response rate between neurons.

It should be noted that the sensitivity of the linear-decoder cannot be directly compared to results from psychoacoustical experiments, as the model only accounts for the lowest stages of the neuronal ITD-detection circuit in gerbils. In other words, it was not the goal of this study to replicate any psychophysical data *per se*, but rather to investigate the performance of the model on its own. Nevertheless, the model could be easily tuned to replicate psychoacoustic threshold data by adjusting the size of the neuronal population to fit human or animal data.

### 3.2. Influence of missing SBCs on the output of the model

In the presented model network, MSO neurons received direct excitatory input from ANFs, while in the physiological case, they receive excitatory inputs from SBCs. SBCs have been found to increase the precision of phase-locking in comparison to ANFs (Dehmel et al., 2010; Künzel et al., 2011). The improvement shown in this study is rather small when compared to the large improvement that has been shown for GBCs (Joris et al., 1994). In spite of this Improvement, the precision is not much higher than that of the ANF model used in this study, and thus no further improvement in phase locking seemed necessary. A second function of SBCs could arise from non-monotonic rate-level functions due to an inhibitory sideband (Künzel et al., 2011; Keine and Rübsamen, 2015). Including a model that would reproduce the non-monotonic rate-level functions may also change the output of the MSO model, specifically, the behavior shown in Figure 5C. This change in the MSOs rate-level function may also be compensated by the ANN, so that the additional feature would not change the message of this paper, leading to the decision to neglect the influence of SBCs. It was also suggested that the slow GABA-ergic inhibition on the level of the SBC may support sound localization of complex sounds by acting as a gain control mechanism (Keine et al., 2016, 2017), this would be interesting to investigate in the context of the presented model but is outside of the scope of this paper.

### 3.3. Performance of the ANN-predictor

The model output showed a strong dependence on both frequency and level of the input signals. Previous models that employed the opponent-coding principle constructed the output of their models to be self normalizing (Pulkki and Hirvonen, 2009; Takanen et al., 2014) or directly extracted the phase from the left and the right input signals using gammatone filters (Dietz et al., 2011). While both methods are valid in view of a phenomenological modeling approach, they can not be easily applied to a neuronal network as presented in this study. We instead showed that a multilayer perceptron could be trained to compensate for frequency and level dependencies and to predict ITD values from the firing rate outputs of the spiking neuron network. By using an ANN to compensate for variability of the MSO output, this study neither makes any assumption about the exact location in the ascending auditory pathway, at which this compensation takes place, nor speculates about the exact mechanism underlying this compensation. We rather show that a very basic ANN containing only twenty hidden nodes in one layer is able to perform the compensation. The ANN-predictor was also shown to provide accurate ITD predictions for complex signals and for time-variant ITDs, even though it was trained on pure tones only. This suggests that the necessary compensation is independent of context. Psychoacoustic studies have shown that sound localization performance depends on the duration (Tobias, 1959) and bandwidth (Trahiotis and Stern, 1989) of the stimulus indicating an integration of information across frequency and time. In this study, the ANN predicted ITDs independently for each frequency and time frame. While integration over the frequency bands was implemented by calculating the mean prediction across all frequencies, no integration over time apart from the calculation of 30 ms averages was performed. Hence, the prediction capability is expected to further improve if the output of the model would also be integrated over time.

While the goal of this study was to evaluate the models' performance on the detection of ITDs, the prime interest of our binaural hearing lies in estimating the direction of a sound source instead of the ITD value. Since low-frequency ITDs between the ear canal signals provide a salient cue about sound source direction, reliable prediction of the ITDs indicates that the azimuthal sound direction may also be accurately predicted. To that end, the ANN could also be trained to directly predict azimuthal angles instead of ITDs.

## 4. Methods

### 4.1. Topology of the model

Both MSO and GBC neurons were modeled using single-compartment, Hodgkin-Huxley-type models simulated in python using the package Brian (Goodman, 2009). MSO as well as GBCs received direct excitatory input from ANF fibers, which were modeled using the model of Zilany et al. (2014), implemented in the python library *cochlea* (Rudnicki et al., 2015). Each population of neurons (ANF, GBC, MSO) always consisted of 500 independent neurons in each hemisphere. The frequency channel of the neuron population was set by selecting the appropriate critical frequency of the peripheral hearing model.

### 4.2. Spiking models

While this study does not discuss the effect of single ionic currents, it makes use of Hodgkin-Huxley-type models, as simpler neuron models like the leaky integrate-and-fire neurons neglect the influence of ion channel dynamics. Especially the shift of best-ITD toward contralateral-leading ITDs has been shown to be influenced by both low-threshold potassium (Myoga et al., 2014) and hyperpolarizing ionic currents (Baumann et al., 2013), both of which are included in this model.

MSO neurons were simulated using single-compartment, Hodgkin-Huxley-type models. The dynamic of their membrane potential *V*_*m*_ is given by the following equation:

(1) $$\begin{array}{l} \frac{dV_{m}}{dt}=-\frac{1}{C_{m}}(I_{leak}+I_{Na}+I_{K}\\ \;+\;I_{h}+I_{syn,e}+I_{syn,i}), \end{array}$$

where *C*_*m*_ is the membrane capacitance, *I*_*leak*_ is the leakage current, *I*_*Na*_, *I*_*K*_, *I*_*h*_ are the sodium, potassium and hyperpolarizing ionic currents and *I*_*syn, e*_, *I*_*syn, i*_ are the excitatory and inhibitory synaptic currents respectively. All ionic currents were defined as follows:

(2) $$\begin{array}{r} I_{x}={ĝ}_{x}a^{m}b^{n}\left(V_{m}-E_{x}\right), \end{array}$$

where ĝ_*x*_ and *E*_*x*_ are the maximal conductivity and Nernst potential for the respective ion species x. The gating variables *a*^*m*^ and *b*^*n*^ determine the channel kinetics. Equations for these variables can be found in the original publication: The sodium dynamics were implemented according to Rothman and Manis (2003) and were corrected for a body temperature of 37 °C (*k* = 3^(*T*−22)/10^). To gain realistic spike shapes as well as a spiking threshold, the activation kinetics had to be sped up by a factor of four. Potassium currents were modeled with the equations for the low threshold channels given by Khurana et al. (2011) with the steady-state inactivation *z*_∞_ set to 0.4. The hyperpolarizing currents were modeled using the equations for dorsal MSO neurons from Baumann et al. (2013). We used a membrane capacity of 70 pF (Couchman et al., 2010) and the ionic conductivities were adjusted to fit the steady state and peak membrane resistances to values measured by Scott et al. (2005). Use of these values resulted in spiking thresholds close to the data published by Couchman et al. (2010). All parameters are summarized in Table 1. GBCs and their synaptic inputs were modeled using the neuron model with 40 non-depressing ANF inputs as proposed by Rudniki and Hemmert (2017).

**Table 1:** Parameters for the MSO model.

**Symbol**	**Value**	**Symbol**	**Value**
*C*_*m*_	70 pF	*E*_*i*_	−70 mV
*E*_*rest*_	−55.8 mV	ĝ_*Na*_	3.9 μS
*E*_*Na*_	56.2 mV	ĝ_*K*_	650 nS
*E*_*K*_	−90 mV	ĝ_*h*_	520 nS
*E*_*h*_	−35 mV	ĝ_*leak*_	13 nS
*E*_*e*_	0 mV		

### 4.3. Synaptic MSO inputs

Each MSO neuron received six excitatory inputs from ANFs of each hemisphere. The excitatory post-synaptic currents (EPSCs) were modeled as an alpha function:

(3) $$\begin{array}{r} I_{syn,e}=\frac{t\cdot e^{1-t/\tau }}{\tau_{e}}\left(V_{m}-E_{e}\right). \end{array}$$

Inhibition was provided via three GBC inputs per hemisphere. The inhibitory post-synaptic currents (IPSCs) were modeled using a bi-exponential function:

(4) $$\begin{array}{r} g_{i}={\hat{g}}_{i}\frac{\tau_{2}\cdot \left(e^{-t/\tau_{i,1}}-e^{-t/\tau_{i,2}}\right)}{\tau_{i,2}-\tau_{i,1}}\cdot \left(V_{m}-E_{i}\right). \end{array}$$

Both, excitatory and inhibitory timeconstants were fitted to recordings by Couchman et al. (2010) yielding values of τ_*e*_ = 0.17 ms and τ_*i*,1_ = 0.14 ms, τ_*i*,2_ = 1.6 ms.

### 4.4. Sound signals and data analysis

All sound signals were generated in Python at a sampling rate of 100 kHz as this sampling rate is required by the peripheral hearing model (Zilany et al., 2014). In the case of the speech signal, the sound was up-sampled from 44.2 to 100 kHz. Each sound signal was gated using a 20 ms long raised-cosine function and 20 ms of silence was attached to the beginning and the end of the signal. The stimuli were presented at a sound pressure level of 50 dB_*SPL*_ if not stated otherwise. ITDs were defined as the difference in the arrival times between the left and the right ears, with positive values corresponding to right leading sounds. To archive sub-sample ITDs, we generated the corresponding delays between the two signals by applying a fast Fourier-transform (FFT), adding the equivalent phase angles, which resulted from the delays, and reverse FFT back to time domain signal.

ITD-rate functions were fitted using a modified Gaussian function as shown in (5) were τ is the ITD value, *R*_*max*_ the maximum firing rate, *W* defines the width of the curve and *B* the location of the maximum (best-ITD).

(5) $$\begin{array}{r} R\left(\tau \right)=R_{max}\cdot e^{\frac{-{\left(\tau -B\right)}^{2}}{W^{2}}}+R_{offset} \end{array}$$

Spiking data were analyzed using the Thorns toolbox for python. Firing rates were always given as the average response of the whole population. To compensate for the intracochlear delay of the inner ear model, we only considered action potentials arriving 25 ms after signal onset and up to 25 ms after the end of the signal.

### 4.5. Calculation of just noticeable differences

JNDs for our model were calculated by presenting two stimuli with ITDs located symmetrically around zero—i.e., −τ/2 and τ/2. The difference between the two ITD was denoted ΔITD . We calculated independently, the difference in firing rate at both hemispheres (ΔR) for each of the presented signals. The two values were then compared to each other. If the ΔR value for the negative ITD signal was larger than the one for the positive ITD signal, the trial was considered as a correct prediction. Each ΔITD was presented 100 times and the fraction of correct trials was calculated. To calculate the JND, we presented 20 logarithmic arranged ΔITD in the range from 2 to 800 μs. The resulting fraction correct values were then fitted with a weibull function. The JND was defined as the ITD at which 75% correct predictions were achieved.

### 4.6. The artificial neural network predictor

The ANN network was implemented using the Theano package for Python. The ANN layout was that of a classic multilayer perceptron containing an input layer with seven nodes, one hidden layers with twenty nodes and an output layer with two nodes (see Figure 6). Both the hidden and the output layer consisted of non-linear nodes with a tanh(*x*) activation function.

The predictor was designed to make predictions for every 30 ms section of the signal. For this, average firing rates for both MSO hemispheres and for the ANF of one hemisphere were calculated in bins of 30 ms. The model firing rates of MSO and ANF as well as the best frequency of these neurons were given as the ANN inputs. To provide some history which can be used to compensate for on- and off-sets, the predictor was also provided with the firing rate in the previous 30 ms bin. Using this information, the ANN gave a prediction of the ITD value in the current bin and a classification whether the presented bin actually contained a signal (signal exists).

The network was trained on the MSO model output from 2,000 different 300 ms long sine tones which were padded by 60 ms of quiet. For each tone the level, frequency as well as ITD were randomly chosen to lie between 30 and 70 dB_SPL_, 125 and 1,000 Hz, and ±500 μs, respectively. The target data for the training set consisted of the ITD value of the corresponding input signal, as well as the classification whether the current time frame contained a signal or not. The target for the classification was set to −1 for the two time bins at the start and at the end of each signal as those contained silence. It was set to 1 for all other bins. The set of training signals was then split into three subsets, a training set containing 80% of the data, a validation and test set both containing 10% of the signals. The ANN was trained on the training set, until the improvement on the mean squared error function for the validation set stayed consistently below 0.01%.

## 5. Data sharing

The neuronal model presented in this paper will be made available on request as well as through the GitHub Repository https://github.com/timtammittee/mso_model_frontiers2017.

## Author contributions

JE: designed the study and wrote the paper; WH: supervised the study and helped with design and writing.

### Conflict of interest statement

The authors declare that the research was conducted in the absence of any commercial or financial relationships that could be construed as a potential conflict of interest.
