# Supplementary material for: Extraction of Inter-Aural Time Differences Using a Spiking Neuron Network Model of the Medial Superior Olive
Source: Front Neurosci. 2018 Mar 6;12:140. doi: 10.3389/fnins.2018.00140 (PMC5845713; doi:10.3389/fnins.2018.00140)
Supplement: Supplementary file 1 [file Image1.PDF]

Supplementary Material: Extraction of inter-aural time differences using a spiking neuron network model of the medial superior olive

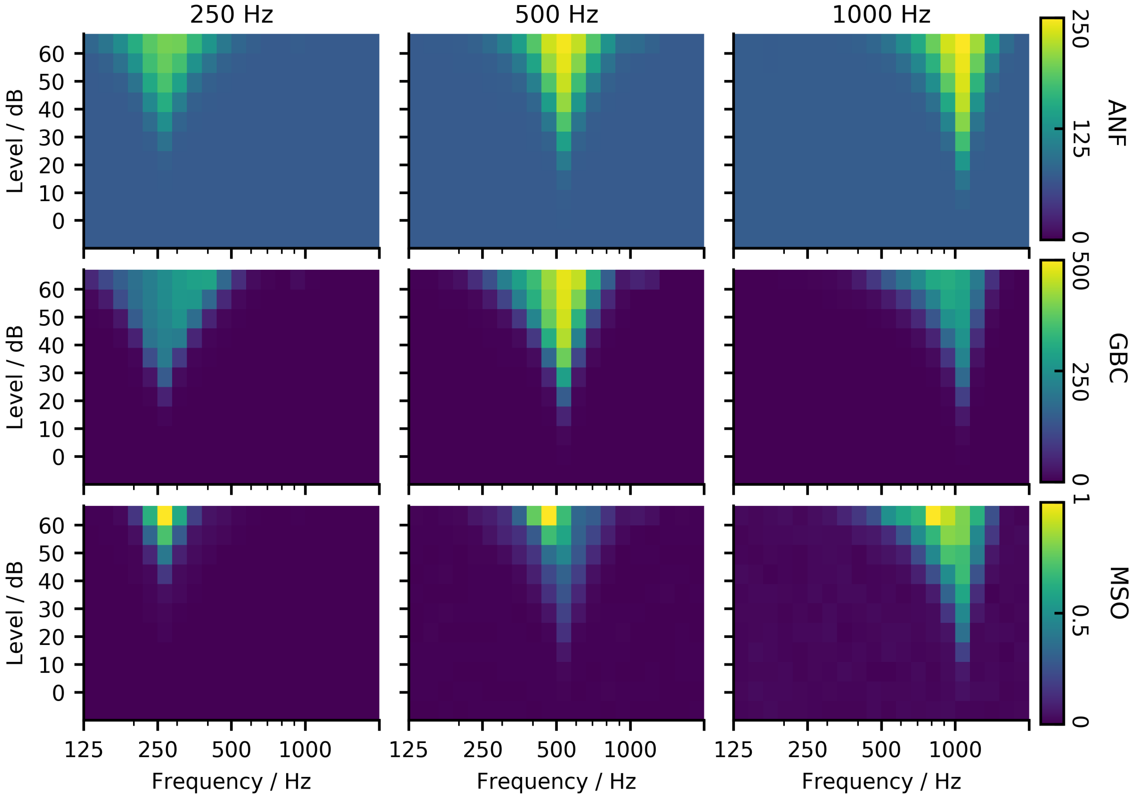

Figure S1: Frequency response areas (FRA) of the three neuronal stages of the model. FRAs were determined by presenting $100\,\mathrm{ms}$ long tone impulses with the frequencies $125\,\mathrm{Hz}$, $250\,\mathrm{Hz}$, $500\,\mathrm{Hz}$ and $1000\,\mathrm{Hz}$ at both ears. The time difference between the two ears was set to $-100\,\mu\mathrm{s}$. Firing rates were calculated as the average rate of the whole population of the right hemisphere. The Firing rates of the MSO population were normalized to account for the frequency dependent sensitivity of the MSO. the normalization factors where 49.7 sps, 132.6 sps, 31.5 sps and 8.7 sps at $125\,\mathrm{Hz}$, $250\,\mathrm{Hz}$, $500\,\mathrm{Hz}$ and $1000\,\mathrm{Hz}$ respectively. As the excitatory input to both, GBC and MSO is the Auditory nerve, they also inherit the shape of the ANFs FRA.

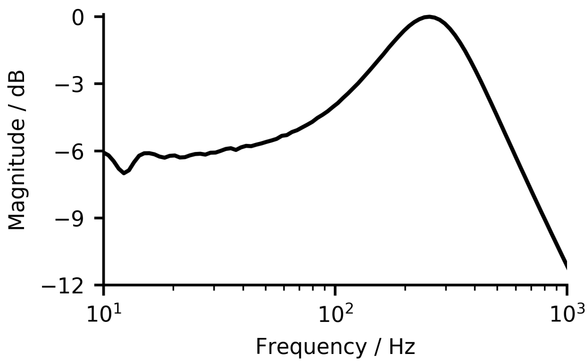

Figure S2: The Frequency response of MSO model shows a second-order filter characteristics with the resonance frequency located at $255\,\mathrm{Hz}$. The Frequency response was calculated by inducing $200\,\mathrm{ms}$ long sinusoidal currents with an amplitude of $100\,\mathrm{pA}$ (cosine window $50\,\mathrm{ms}$ rise time) and determining the amplitude of the membrane potential.
